# Enteric Hard Capsules for Targeting the Small Intestine: Positive Correlation between In Vitro Disintegration and Dissolution Times

**DOI:** 10.3390/pharmaceutics12020123

**Published:** 2020-02-03

**Authors:** Maoqi Fu, Jozef Al-Gousous, Johannes Andreas Blechar, Peter Langguth

**Affiliations:** Department of Biopharmaceutics and Pharmaceutical Technology, Johannes Gutenberg University Mainz, D-55099 Mainz, Germany; maoqifu1@uni-mainz.de (M.F.); joalgous@uni-mainz.de (J.A.-G.); jblechar@uni-mainz.de (J.A.B.)

**Keywords:** disintegration, dissolution, enteric-coated, ICH, quality control

## Abstract

In this study, the potential for correlation between disintegration and dissolution performance of enteric-coated (EC) dosage forms was investigated. Different enteric hard shell capsule formulations containing caffeine as model drug were tested for disintegration (in a compendial disintegration tester) and for dissolution in both USP type I (basket) and type II (paddle) apparatuses using different media. Overall, good correlations were obtained. This was observed for both the basket and the paddle apparatus, indicating that the use of disintegration testing as a surrogate for dissolution testing (allowed by International Conference on Harmonization (ICH) for immediate release dosage forms in case, in addition to other conditions, a correlation between disintegration and dissolution is proven) could be extended to include delayed release dosage forms.

## 1. Introduction

Disintegration tests have been used to evaluate dosage form performance since the early 20th century, with the current compendial disintegration tester being available since the 1950s [1]. Despite the limitation of the released amount of the active pharmaceutical ingredient (API) not being measured, these tests are still widely used in pharmaceutical practice owing to their simplicity and speediness compared to dissolution tests. This contributed to the International Conference on Harmonization (ICH) allowing disintegration tests to be used as dissolution test surrogates if (among other conditions) a correlation between disintegration and dissolution is proven [2]. 

In this regard, it appears from the literature data that the likelihood of obtaining a disintegration–dissolution correlation varies greatly from formulation to formulation, with Gupta et al. [3] testing a 12 different verapamil hydrochloride formulations and finding that only one of them gave a satisfactory correlation. Radwan et al. [4] investigated different trospium chloride formulations under different conditions and found that a correlation is possible when the disintegration is not too rapid. Nickerson et al. [5] on the other hand found good correlations for several immediate formulations of an unnamed API. However, the focus has generally been restricted to immediate release dosage forms, most probably because of the ICH guidance restricting the possibility of employing disintegration test as a dissolution test surrogate to non-modified release dosage forms.

However, one class of modified release dosage forms, namely enteric-coated (EC) formulations, offers at least a theoretical possibility for obtaining good disintegration dissolution correlations. For, in the presence of a rapidly disintegrating and dissolving core, having a situation where the disintegration of the enteric coat strongly influences the overall release performance is likely. Therefore, this work is going to investigate the correlation between disintegration and dissolution of enteric-coated hard shell capsules in order to explore the feasibility of employing the disintegration test as a dissolution test surrogate for EC dosage forms.

## 2. Materials and Methods

### 2.1. Materials

Hydroxypropyl methylcellulose size 0 capsules (ACG Nature Caps Plus) were received form ACG Associated Capsules Pvt Ltd (Mumbai, India). DRcaps^®^ (nutraceutical capsules with inherent enteric properties of the capsule shell) were obtained from Neue Lebensqualität (Badendorf, Germany). Caffeine (median particle size 48 µm) and magnesium stearate were purchased from Caesar & Loretz GmbH (Hilden, Germany). Fumed silica was purchased from Fagron GmbH & CO.KG (Glinde, Germany). Hypromellose phthalate (HP-50), hypromellose acetate succinate (HPMCAS-HG, AQOAT) and low substituted hydroxypropyl cellulose (L-HPC) were received as a gift from Shin-Etsu (Wiesbaden, Germany). Lactose (FlowLac^®^ 90) was received from Molkerei Meggle (Wasserburg, Germany). Triethyl citrate (TEC) was purchased from Sigma-Aldrich (Overijse, Belgium). Talc was purchased from Imerys (Luzenac, France). All other materials were of analytical grade.

### 2.2. Capsule Filling

The HPMC capsules and DRcaps^®^ were filled manually (aponorm^®^ Kapselfüllgerät, WEPA Apothekenbedarf GmbH & CO.KG, Hillscheid, Germany) with a powder formulation. The powder formulation was prepared using a 1.6 L Turbula mixer (Willy A. Bachofen GmbH, Muttenz, Switzerland) at 49 rpm (batch size 600 g), and its composition is described in Table 1. The capsules were filled with 375 mg of the powder (i.e., 75 mg of caffeine). The filled capsules complied with the content uniformity requirements of the European Pharmacopoeia 9.0.

### 2.3. Capsule Coating

The ACG Nature Caps Plus were coated. Two different batches of coated capsules were prepared from them. The coating formulations are described in Table 2. First, the ethanol–water solution was prepared. The polymer was dissolved in 80% of the solvent and the remaining 20% of the solvent were used to disperse the talc. Afterwards, the polymer solution is combined with the talc dispersion. Last, triethyl citrate is added to the formulation (in case of the HPMCAS-HG formulation). Before coating, the polymer solution is filtered using a sieve with a pore size of 0.2–0.4 mm. The coating levels of the capsules coated with HP-50 and HPMCAS-HG are 10 mg polymer/cm^2^ and 9 mg polymer/cm^2^ (30% and 27% weight gain), respectively. A Solidlab 1 drum coater (Robert Bosch Packaging Technology GmbH, Waiblingen, Germany) was used for coating with the following parameters: 230 g of capsules/batch preheated to 30 °C; spray rate of 6.5–7 g/min and a atomizing pressure of 2.0 bar; nozzle diameter of 0.5 mm; the inlet air was heated to 58–60 °C and had a flow rate of 55 m^3^/h; product temperature of 35–38 °C.

### 2.4. Disintegration Test

The capsule disintegration was performed with disks using a DT2 Disintegration Tester (Sotax AG, Aesh, Switzerland) complying with the European Pharmacopoeia specifications for a type A disintegrat ion testing apparatus. All capsules were first exposed to 700 mL of 0.1 M HCl for one hour followed by one hour testing in 700 mL of buffer. The temperature was maintained at 37.0 ± 0.5 °C There were three different buffers tested, namely the 50 mM USP phosphate buffer pH 6.8, blank FaSSIF buffer (28.4 mM) pH 6.5 and a 15 mM phosphate buffer pH 6.5 that showed to be biopredictive in previous studies (henceforth referred to as the “Al-Gousous et al. medium” [6]). The disintegration times recorded are the times at which the capsules ruptured, which helps reduce the uncertainty associated with determining the disintegration times based on “complete disintegration” [7]. Accordingly, these times are defined as the times at which first visible cracks in the capsule shell appear. In order to avoid observer bias, disintegration tests were performed before dissolution tests.

### 2.5. Acid Uptake Test

Six capsules were individually weighed and then tested in a disintegration tester as outlined above (the Disintegration Test subsection) but without disks, and only in HCl (0.1 and 0.01 M) for one and two hours. Sinkers (Japanese Pharmacopoeia Standard, Pharma Test, Hainburg, Germany) were used to prevent the capsules from floating. At the end of the test the capsules were removed, blotted and the %weight gain was calculated as follows:

$$\%\;\mathrm{weight}\;\mathrm{gain}\;\mathrm{in}\;\mathrm{acid}=\frac{\mathrm{mass}\;\mathrm{after}\;\mathrm{acid}\;\mathrm{exposure}-\mathrm{mass}\;\mathrm{before}\;\mathrm{acid}\;\mathrm{exposure}}{\mathrm{mass}\;\mathrm{before}\;\mathrm{acid}\;\mathrm{exposure}}\times 100\%$$

### 2.6. Dissolution Test

The drug release was tested with a DT6R dissolution tester (Erweka GmbH & CO.KG, Langen, Germany). The device was used as a USP type I dissolution tester at 100 rpm as well as a USP type II dissolution tester with sinkers (same as in previous subsection) at 50 rpm. The use of sinkers prevented the capsules from floating in the paddle apparatus. In accordance with the disintegration test, the capsules were studied for one hour in 0.1 M HCl followed by a media change to either one of the buffers described previously. The volume of the dissolution media was 700 mL. The temperature was maintained at 37.0 ± 0.2 °C. The 5 mL samples were filtered through a 0.8 µm cellulose acetate nitrate filter (Rotilab Spritzenfilter CME, Carl Roth, Karlsruhe, Germany). The first 1 mL of the filtrate was discarded to saturate the membrane. Blank buffer was used to replace the sample volume. Caffeine was quantified spectrophotometrically at λ = 275 nm. 

### 2.7. Correlation between Disintegration and Dissolution Results

Disintegration times were correlated with the times required to achieve 10%, 50% and 80% release (t10%, t50% and t80% respectively) representing the early, middle and late portions of the dissolution profiles. The aforementioned times were calculated using linear interpolation. The correlation was done using simple linear regression performed by Microsoft Excel (Microsoft Office 2013, Microsoft Corporation, Redmond, WA, USA). Hypothesis testing was performed on the slope using a one-sided *t*-test (With the null hypothesis being slope = 0). One-sided *p*-values were calculated since a positive correlation is expected. The hypothesis testing was performed using the vassarstats website [8].

## 3. Results and Discussion

### 3.1. Disintegration and Acid Uptake

**Figure 1:**
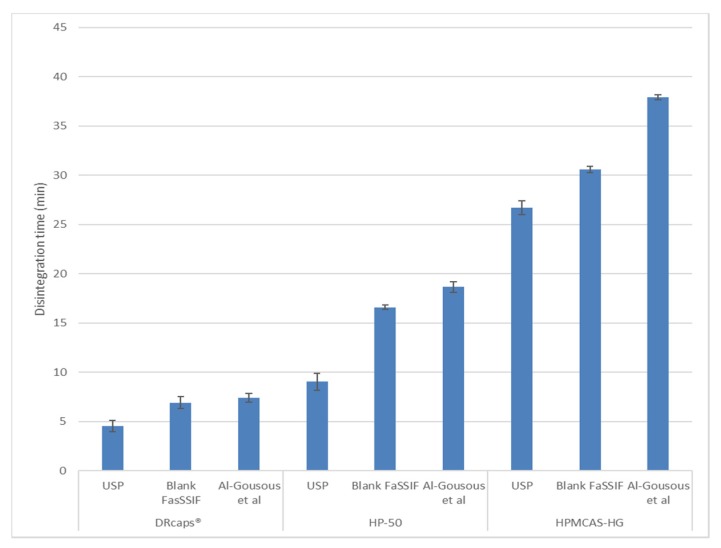
Disintegration times (mean ± SD) of the tested formulations (n = 6).

**Figure 2:**
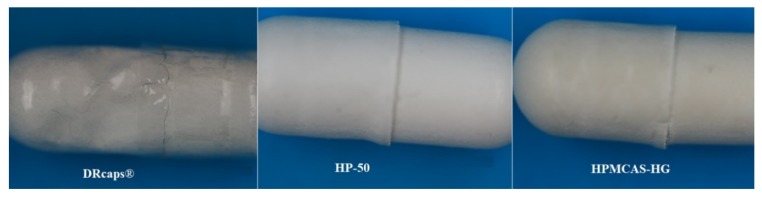
Appearance of DRcaps® and coated capsules after 1 h in 0.1 M HCl.

As shown in Figure 1, DRcaps® Enteric gave the fastest disintegration while HPMCAS-HG gave the slowest. Disintegration tended to be fastest in the USP dissolution testing medium and slowest in the Al-Gousous et al. medium as would be expected based on the buffer molarities of the media. The fast disintegration of DRcaps® Enteric, however, seems to be associated with poor resistance to acid as evidenced by the acid uptake values shown in Table 3 and by the deformation exhibited by those capsules (Figure 2). This indicates that it is rather the weakened capsule shell structure that results in rapid disintegration in buffer. This is rather in line with the findings of Al-Tabakha et al. [7], where even rupture of such capsule shells was observed in simulated gastric fluid.

It is interesting that despite showing the lowest acid uptake at one hour, the HPMCAS-HG capsules ruptured in acid within roughly 1.5–2 h during the acid uptake tests. This might be related to mechanical instability. Figure 2 shows that while the DRCaps® show extensive deformation, HP-50-coated ones show only some wear at the gap between the body and the cap. HPMCAS-HG-coated capsules behave in a manner similar to the HP-50-coated ones but the wear at the gap seems to be a bit more extensive, which may impart mechanical instability to the capsule. The causes behind this need to be further investigated.

As shown in Figure 1, after 1 h in acid, the capsules coated with HPMCAS-HG still show the longest disintegration times. Only the high buffer capacity USP medium [6] showed large acceleration in disintegration compared to the situation where testing in acid was continued for one further hour (in the acid uptake tests with sinkers and without disks). This further supports the mechanical instability hypothesis. As for why the presence of disks in the disintegration test (compared to their absence in the acid uptake test) does not seem to have a dramatic effect, this might be related to the disk impacting the capsule from above rather than tearing it apart. Other factors could be the force generated on contact between the capsules and the sinkers in the disk-free setup as well as the tilted orientation of the capsules in the disintegration tester tubes when inside sinkers and its potential effects on hydrodynamics. Further investigation is needed regarding this issue, which is outside the scope of this manuscript.

### 3.2. Dissolution

**Figure 3:**
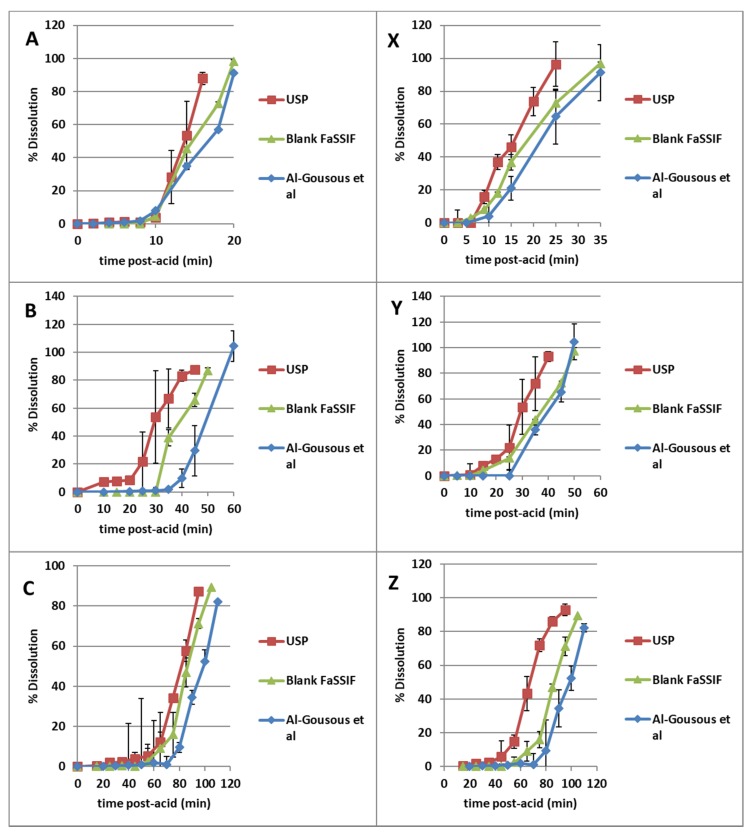
Dissolution test results (mean ± SD) of the tested formulations (n = 6). Panels (**A**–**C**) represent DRcaps®, HP-50 and HPMCAS-H respectively in basket apparatus, while panels **X**, **Y** and **Z** represent DRcaps®, HP-50 and HPMCAS-H respectively in paddle apparatus.

As shown in Figure 3, the dissolution results followed the trends exhibited by the disintegration times. The disintegration times are lesser even than the t10% values, which is most probably associated with the capsule rupture being a pre-requisite for significant drug release and with the greater mechanical stresses in the disintegration tester [9]. Dissolution tended to be slower in the basket apparatus. This may be associated with the lower fluid velocities in the central and upper regions of the basket (at 100 rpm) where the capsule tends to be located owing to its buoyancy compared to the bottom of the vessel in a paddle apparatus (at 50 rpm) [10]. This implies that hydrodynamic differences between the different apparatuses play an important role in the obtained correlations.

### 3.3. Correlation between Disintegration and Dissolution Results

**Figure 4:**
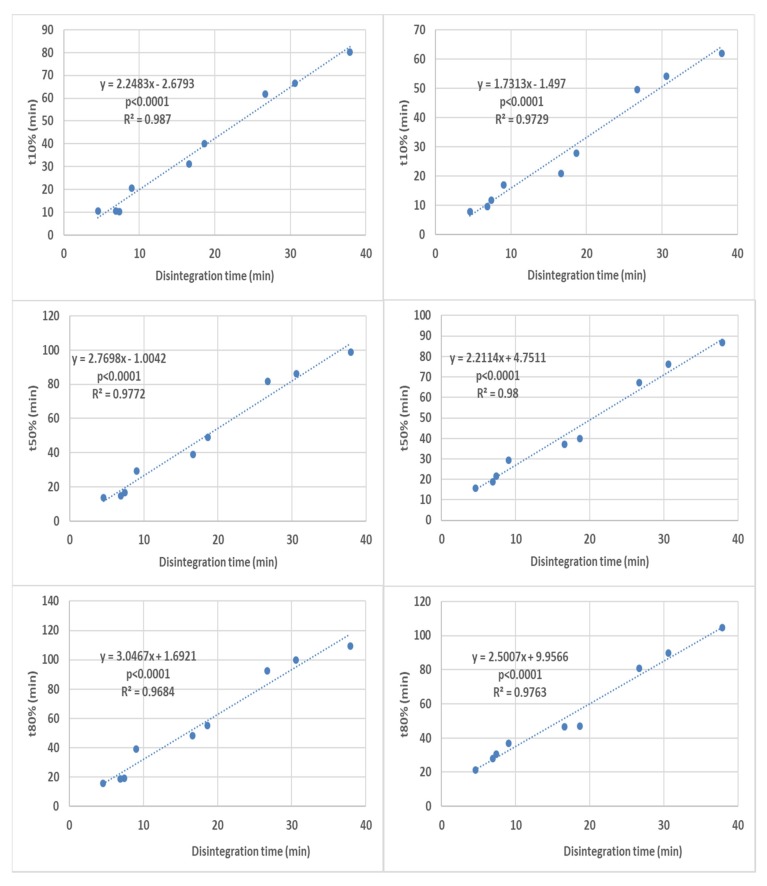
Overall correlation of all the disintegration results with their respective dissolution results (all formulations in all media are present in each graph) in the basket apparatus (right-hand side) and the paddle apparatus (left-hand-side). The *p*-value is a one-sided value for a *t*-test applied to the slope.

Disintegration times were correlated with the times required to achieve 10, 50 and 80% release (t10%, t50% and t80% respectively) representing the early, middle and late portions of the dissolution profiles. When all the dissolution times were correlated with their respective disintegration times, good overall correlations were obtained for all dissolution profile portions (Figure 4). 

**Figure 5:**
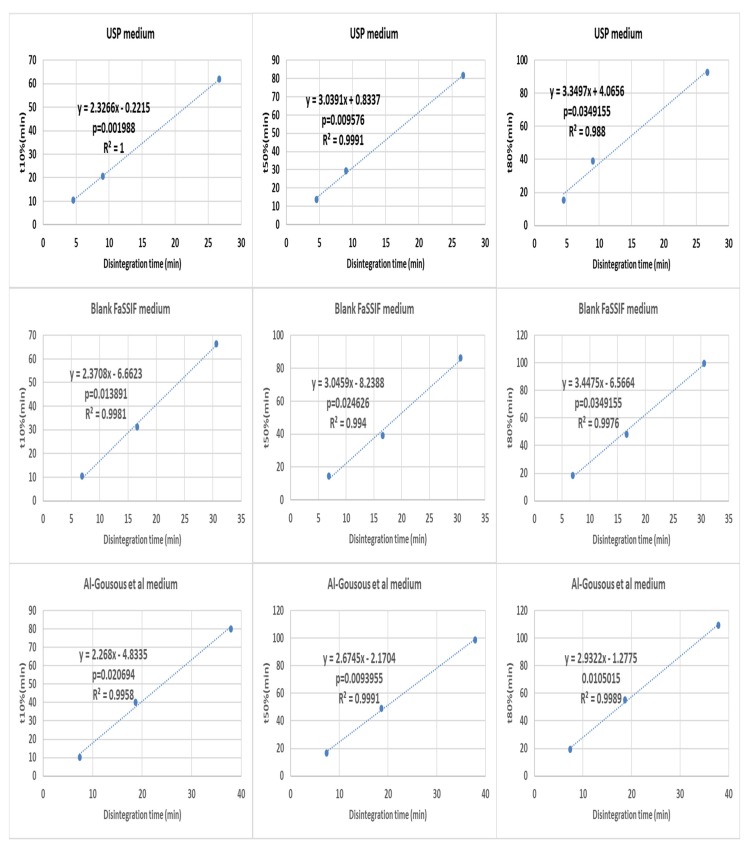
Correlating disintegration times with different dissolution parameters (basket apparatus) for different formulations tested in one medium. The *p*-value is a one-sided value for a *t*-test applied to the slope.

**Figure 6:**
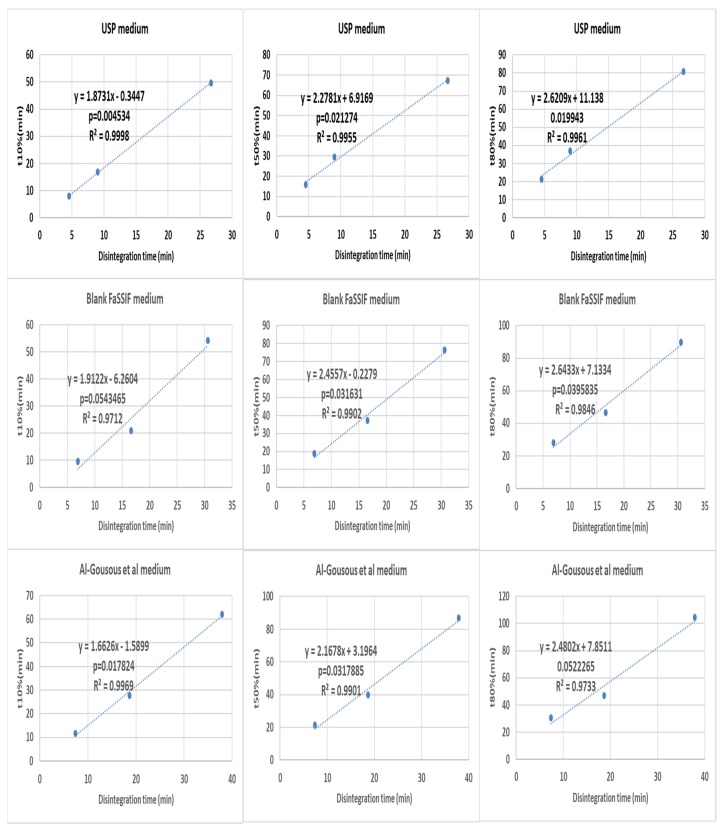
Correlating disintegration times with different dissolution (paddle apparatus) parameters for different formulations tested in one medium. The *p*-value is a one-sided value for a *t*-test applied to the slope.

**Figure 7:**
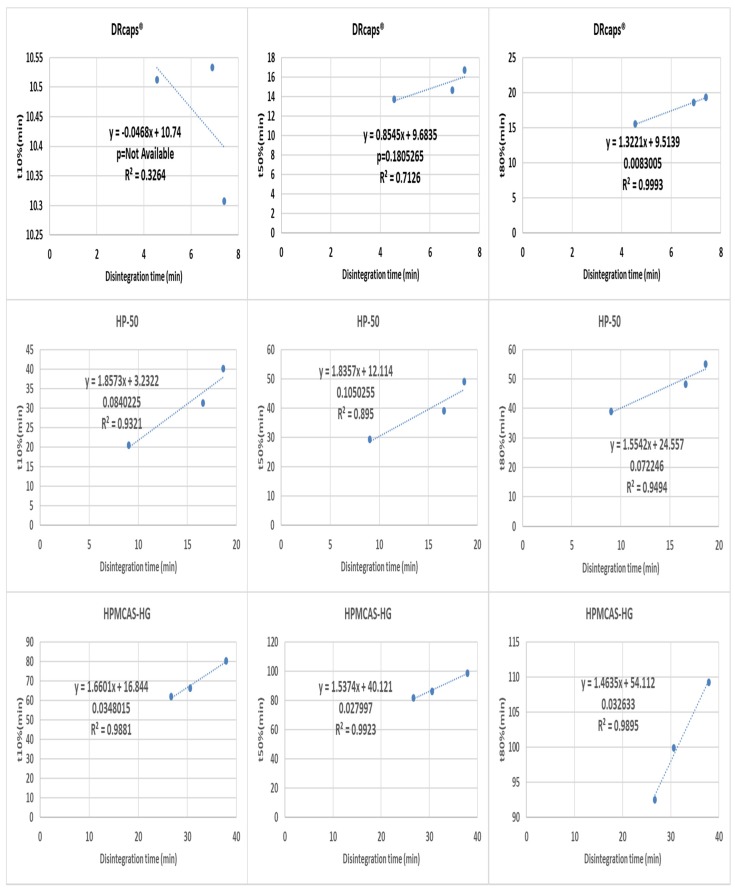
Correlating disintegration times with different dissolution (basket apparatus) parameters for one formulation in different media. The *p*-value is a one-sided value for a *t*-test applied to the slope.

**Figure 8:**
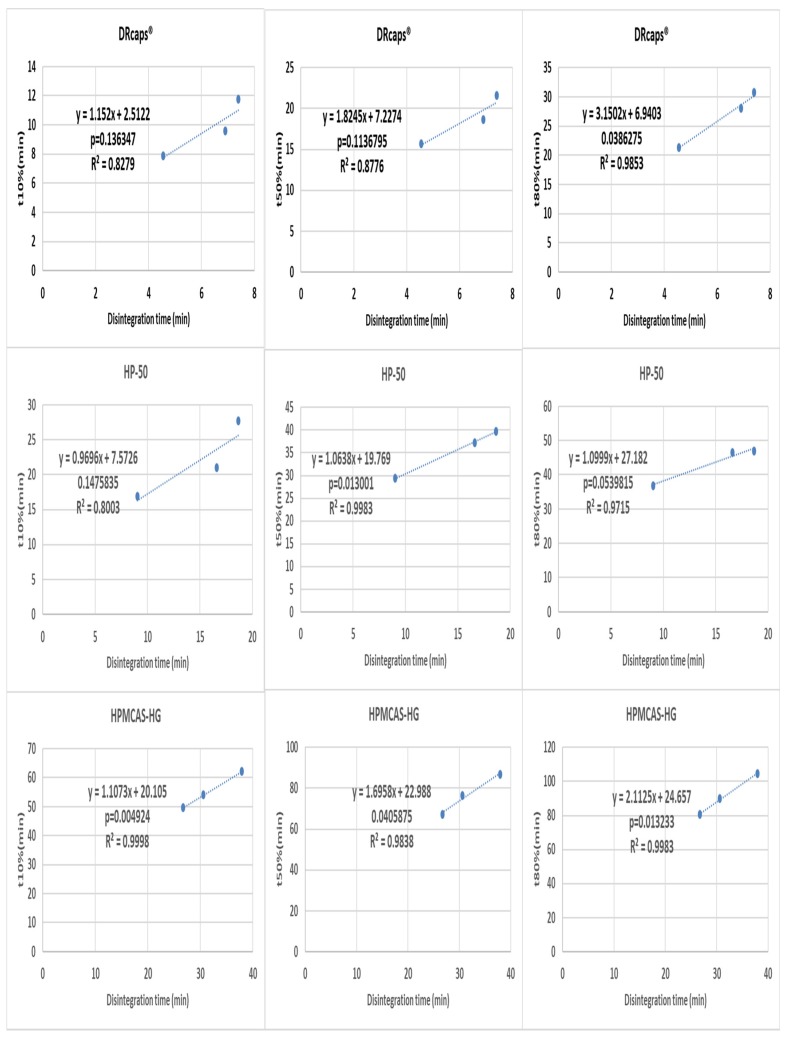
Correlation of disintegration times with different dissolution (paddle apparatus) parameters for one formulation in different media. The *p*-value is a one-sided value for a *t*-test applied to the slope.

**Figure 9:**
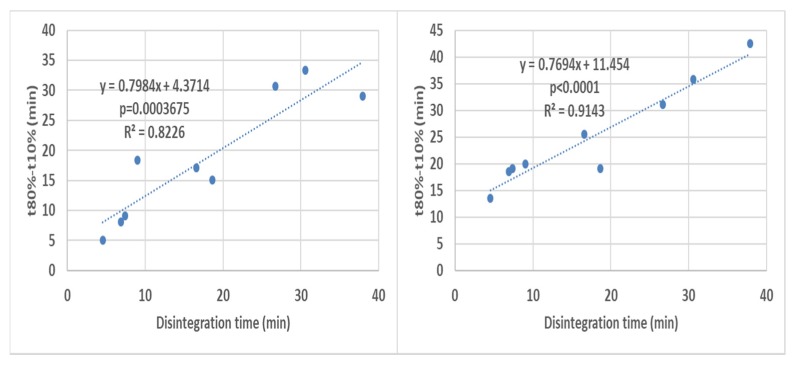
Overall correlation of all the disintegration times with their (t80%–t10%) values results (all formulations in all media are present in each graph) in the basket apparatus (right-hand side) and the paddle apparatus (left-hand-side). The *p*-value is a one-sided value for a *t*-test applied to the slope.

A more detailed analysis was performed by making three point correlations for formulation effects (Figure 5 and Figure 6) and medium effects (Figure 7 and Figure 8). When the results of different formulations tested in the same medium were correlated, good r^2^ and p-values were almost invariably obtained. However, the situation was different when correlating results of the same formulation in different media (Figure 8 and Figure 9), where the differences tended to be smaller than the inter-formulation differences. 

Poor correlations were generally obtained for DRcaps® (with the notable exception of the t80% case). A possible explanation for that could be that the weakened capsule shell structure made the initial rupture more associated with random mechanical events and less with the enteric-polymer dissolution promoting capabilities of the buffer. The complete process of shell dissolution/disintegration was less confounded by such random effects resulting in better correlations for t80%. As for the weak correlations obtained for the t10% and t80% parameters for the HP-50-coated capsules, they seem to be caused by the close disintegration times in the blank FaSSIF and Al-Gousous et al. media. This is most probably related to the different discriminative abilities of the disintegration tester vs. paddle and basket apparatuses (owing to the different hydrodynamics). HPMCAS-HG gave the best correlations likely due to the slow capsule shell disintegration relative to drug dissolution. Anyway, despite multiple instances of weak correlations, when correlating the results of one formulation in different media, each formulation shows at least one instance with *p* < 0.05. 

An additional observation is that the correlations for different dissolution time points show not only different intercepts but also different slopes. This indicates that the disintegration times do not correlate with the different dissolution times solely because of the profiles being shifted because of different coat rupture times, but also because of the influence of the disintegration on the overall post-capsule rupture release kinetics. This is shown by the fair to strong overall correlations obtained for the difference between t80% and t10% (corresponding to the time required for % release to rise from 10% to 80%).

Figure 9 shows stronger correlation for the paddle apparatus. A possible reason could be that variation in the floating capsule orientation inside the basket, together with the more variable fluid velocities in the upper region of the basket [10], leads to the greater data point scatter observed for the basket apparatus. 

All in all, the obtained set of correlations shows that enteric-coated formulations seem promising with regard to using disintegration tests as dissolution surrogates. This shows that the use of disintegration testing as dissolution testing surrogate might not have to be restricted only to immediate release dosage forms. However, further investigations on further EC dosage forms need to be performed before making a definitive judgment on this matter. 

## 4. Conclusions

Obtaining good correlations between the dissolution and disintegration results of EC dosage forms is possible. This opens the way for more rigorous research that could help in expanding the dissolution test waiver concept beyond immediate release dosage forms. Further investigations on additional EC formulations could help to establish regulatory criteria regarding this matter. However, extrapolating these findings to the in vivo situation should be done with extreme caution since many factors like (among others) possible transporter saturation effects [11], interplay with food and gastric emptying effects [12,13] as well as different hydrodynamics and mechanical stresses [9] complicate the correlation between disintegration and bioavailability. These issues need to be taken into account when considering an expanded role for disintegration testing in product evaluation.

## Figures and Tables

**Table 1:** Formulation of the capsule filling. All values (%) are based on the total weight (m/m).

Substance	% (m/m)	Weights of the Components per Capsule (mg)
Caffeine	20	75
L-HPC	15	56.25
Lactose	63.75	239.06
Silica	0.25	0.94
Magnesium stearate	1	3.75
Total	100	375

**Table 2:** Coating formulation. All values (%) are based on the total solution.

Substance	HP-50 Formulation		HPMCAS-HG Formulation	
	% (m/m)	Weights of the Components (g)	% (m/m)	Weights of the Components (g)
Polymer	6	60	5	40
Talc	7.5	75	7.5	60
TEC	-	-	2	16
Ethanol	69.2	747.7	68.4	591.25
Water	17.3	117.3	17.1	92.75

**Table 3:** Weight gain (mean ± SD) of the tested formulations (n = 6) after 1 h in acidic media.

Formulation	% Weight gain in 0.1 M HCl		% Weight gain in 0.01 M HCl	
	After 1 h	After 2 h	After 1 h	After 2 h
DRcaps®	6.5 ± 0.7	7.4 ± 0.3	11.2 ± 0.2	13.5 ± 0.6
HP-50	3.4 ± 0.6	4.2 ± 0.3	3.8 ± 0.1	6.1 ± 0.7
HPMCAS-HG	2.5 ± 0.4	Ruptured	2.8 ± 0.6	Ruptured
